# The Anticipated Organ Donation Approach Increases the Number of Organ Donors

**DOI:** 10.4274/TJAR.2024.241676

**Published:** 2024-12-16

**Authors:** Erwan d’Aranda, Valérie Arsonneau, Didier Demory

**Affiliations:** 1 Sainte Anne Military Teaching Hospital, Intensive Care Unit, Toulon, France; 2 CHITS Sainte-Musse Hospital, Local Organ Procurement Organization, Toulon, France; 3 CHITS Sainte-Musse Hospital, Intensive Care Unit, Toulon, France

**Keywords:** Brain death, intensive care, organ donors, organ procurement, tissue donors

## Abstract

**Objective:**

Deficiency of organs for transplantation is a significant public health issue. French learned societies accept intensive care unit admission for patients with catastrophic neurological prognosis to optimize organs prior to probable brain death. We evaluated the implementation of a specific ethical care procedure for these patients.

**Methods:**

A descriptive before-after study was conducted, comparing the 2009-2012 period to the 2013-2016 period, during which this procedure was applied.

**Results:**

The number of patients increased from 145 to 186 (+28.3%) and the number of harvested organs increased from 323 to 485 (+50.1%). The anticipated organ donation approach was initiated 135 times. Of the 117 meetings with families, 83 (71%) consented to organ donation. Fifty-three (64%) patients were brain dead, and 49 (92%) of these patients had 194 organs harvested.

**Conclusion:**

The anticipated approach increased the number of donors and organs suitable for grafts. The application of a specific protocol for managing untreatable catastrophic neurological patients improved communication between hospital staff and families and respected patient autonomy by offering options for either organ donation or palliative care.

**Main Points**

• Implementing a specific protocol for managing catastrophic neurological patients resulted in an increase in actual donors and organ donors.

• This protocol respects patients’ autonomy and facilitates relationships with their families.

• This protocol could help reduce the shortage of transplant recipients.

## Introduction

The shortage of transplanted organs is a significant medical and social issue, as transplantation is often the best therapeutic option for end-stage organ failure.^1^ One donor has the potential to impact the lives of six others. Organ donation is a national priority in France, with several “graft plans” implemented by the French Bio-medicine Agency, the national organ procurement organization (OPO), to increase public and healthcare staff awareness.^2^ There is no age limit for organ donors, and with improved medical management, the criteria for potential organ donor selection have been expanded since 2003.^3^ In 2016, 9.9% of the donors were alive, 90.1% were deceased, and 95.2% were brain dead.^4^ Over the past 20 years, the etiology of death has changed, with traumatic brain injuries accounting for over 50% of deaths in the 1990s and over 60% of deaths today attributed to stroke. In 2016, 3,676 patients were identified as potential organ donors, but only 1,770 patients became actual organ donors, resulting in 5,891 solid organ transplantations. Although impressive, the annual number of organ transplants is low compared with the 15,465 patients on the waiting list, which continues to grow.^4^ Patients with renal failure are the most numerous on the waiting list, and they are increasingly older, which decreases their chances of transplantation. An increase in potential donors is necessary.

Given the lack of grafts and longer waiting lists, the use of marginal transplants has increased, particularly in elderly patients. Intensive care unit (ICU) admission of patients with catastrophic neurological prognosis presents ethical and economic challenges for ICU teams. The legitimacy of this care was established in 2010. French intensivist approved ICU admission of patients with severe coma after infarction or cerebral hemorrhage in the absence of therapeutic resources and when progression toward brain death is likely for exclusive organ donation.^1, 5^ This is an anticipated organ donation approach. Implementation in hospitals requires new practices to identify potential donors, select patients, and communicate with families. We evaluated the implementation of a specific ethical care procedure for patients with catastrophic brain injuries.

## Methods

The study protocol consisted of a retrospective analysis of an anonymised database without any direct human involvement. In accordance with the French law at the time of the study, the protocol received approval (approval number: 8.4.17, no.: 376) from the local ethics review board on April 8, 2017, at the military teaching hospital Sainte Anne, Toulon, France.

We conducted a retrospective cohort study in a local OPO located in Centre Hospitalier Intercommunal de Toulon, France, which works with four regional hospitals. We compared two periods: 2009-2012, during which an anticipated organ donation approach was not in place, and 2013-2016, during which a specific protocol was initiated. We included all patients diagnosed with complete brain death or who were included in the anticipated organ donation approach. The collected data included demographics (age), number of potential organ donors, number of actual organ donors, number and type of organs retrieved, and evolution after identification of potential organ donors or after inclusion in the anticipated organ donation approach.

### Anticipated Organ Donation

**Figure 1:**
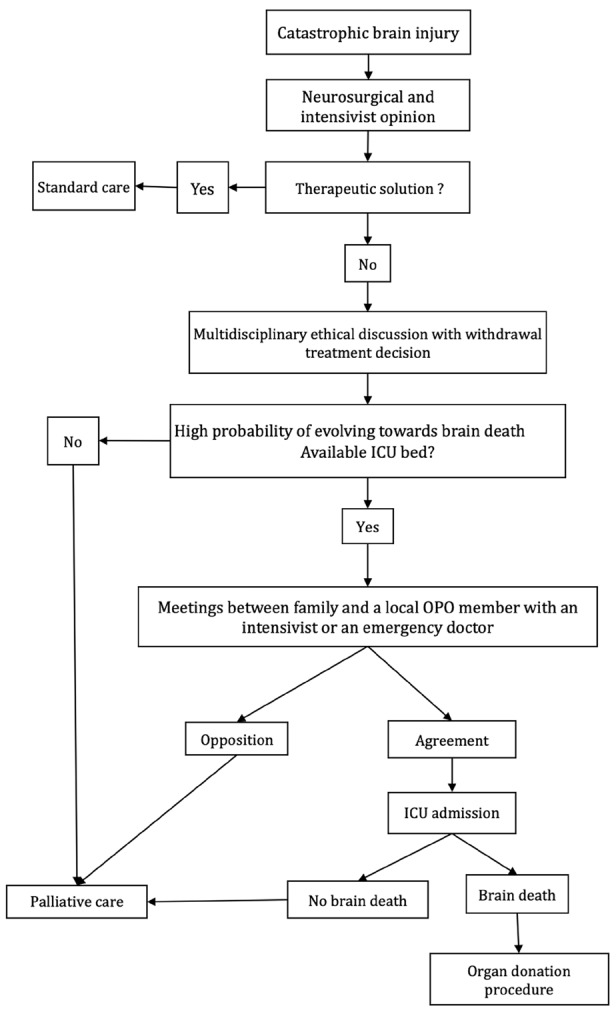
Step-by-step screening of a patient with catastrophic brain injury.

The aim of this approach is to admit patients to the ICU who do not have brain death but have a high probability of subsequent brain death. These patients require a specific protocol to avoid initiating an anticipated procedure if there is an obvious obstacle to organ donation, if the patient cannot be medically treated (e.g., no beds available in the ICU), or if the patient is not progressing toward brain death. We performed step-by-step screening in the emergency unit, stopping the procedure when any step resulted in a “no” (Figure 1). We screened patients with catastrophic brain injuries after infarction or cerebral hemorrhage, in an absence of therapeutic resources, and after multidisciplinary ethics discussions to make treatment withdrawal decisions.

The local OPO was contacted to coordinate the protocol. We evaluated whether the patient had a high probability of progressing toward brain death. We used locally defined criteria to identify a brain state preceding the “imminent brain death” state by 24-48 hours. These selection criteria evolved during the implementation of the procedure and included either a Glasgow coma score <5 without confounding factors and the absence of bilateral corneal reflex or the disappearance of three brainstem reflexes; an initial Glasgow coma score <7 with at least one negative element on computed tomography: obliterated basal cisterns, subfalcine herniation superior to 15 mm, hematoma superior to 65 mL, or intraventricular hemorrhage with bleeding inside V3 or V4.

We subsequently evaluated whether the patient was a potential organ donor by reviewing their medical and surgical history, performing a clinical examination, and assessing kidney and liver function. If a bed was available in the ICU within 6 hours, the patient was included in the anticipated approach. The family was contacted to confirm that there was no opposition to organ donation under French law. A local OPO member met with an intensivist or an emergency doctor. Patients without opposition were admitted to the ICU for nontherapeutic care, which only included organ preservation and patient comfort. We allowed 48 hours for brain death diagnosis. For patients whose families objected to organ donation, or if there were no free beds in the ICU or no brain death after 48 hours, palliative care was initiated. The local OPO member established a moral contract with the family to inform them and make decisions within a reasonable timeframe.

### Management of Potential Organ Donors

The management of potential organ donors was guided by international guidelines.^2, 6^ In the absence of evidence of cortical function and brainstem reflexes with no confounding factors, an apnea test was performed with continuous positive airway pressure at 10 cm H_2_O. The ancillary testing methods for determining brain death were cerebral angiography and electroencephalography.

### Statistical Analysis

Quantitative data are provided as numbers, means (standard deviation), or percentages. All analyses were performed with Excel 2011 (Microsoft, USA).

## Results

**Figure 2:**
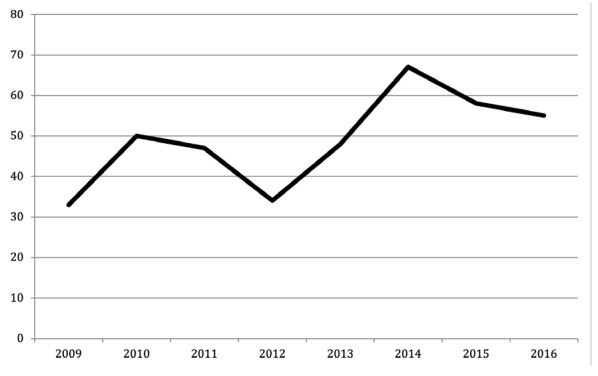
Number of renal grafts per year.

We identified 331 potential organ donors with brain death over 8 years. Of these, 210 became actual donors, resulting in 808 organs being transplanted. Table 1 presents the number of organ donors during the first and second periods and the anticipated approach during the second period. A mean of 36.5 potential organ donors per year before 2012 and 46.5 per year beginning in 2012 represented a gain of 28.3%. The number of actual organ donors increased from 22.75 per year to 29.75 per year, representing a gain of 30.7%. The number of solid organs transplanted increased by 50.1% from a mean of 80.75 transplantations per year to 121.25 transplantations per year. The number of annual renal grafts is shown in Figure 2.

**Figure 3:**
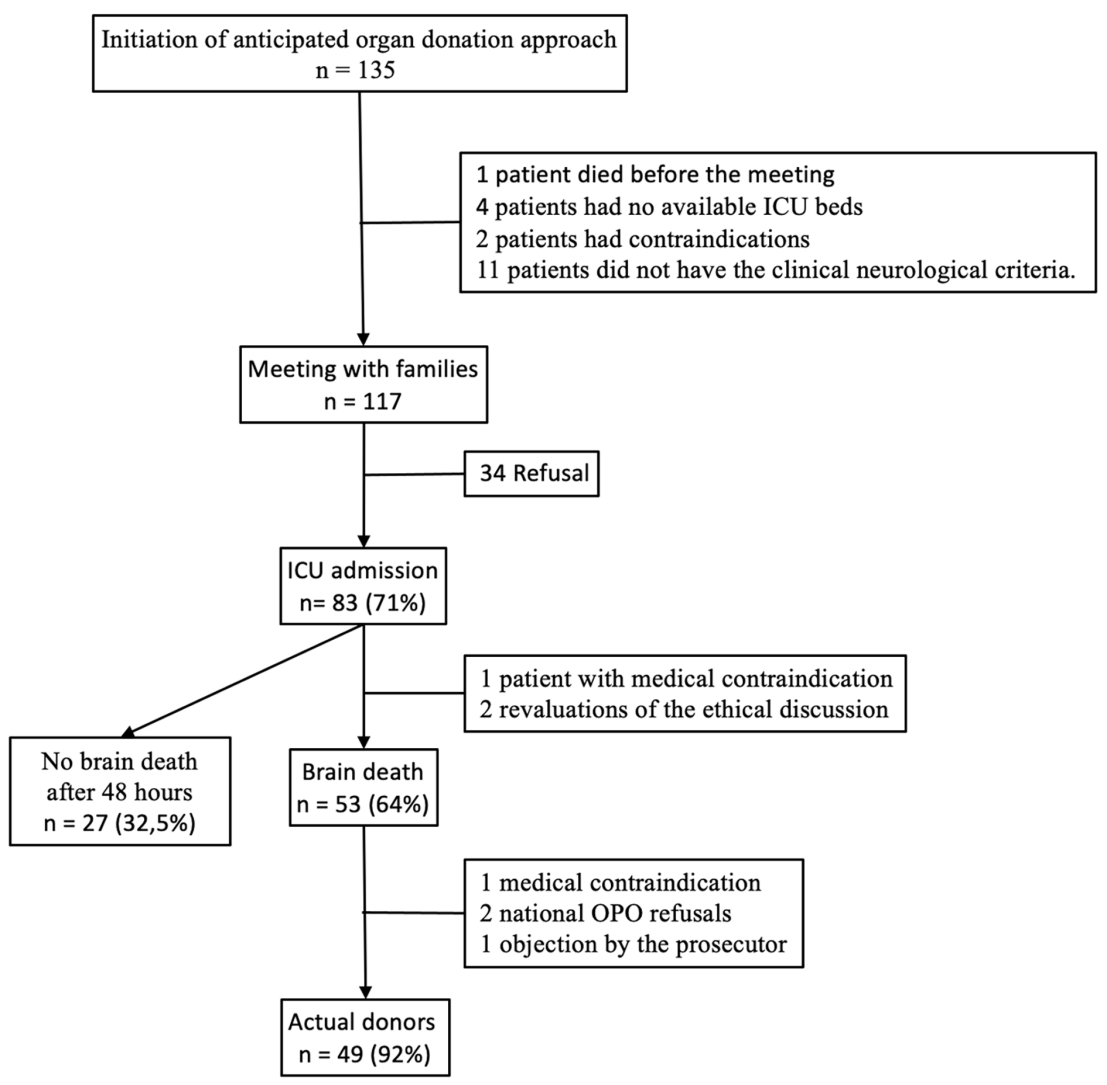
Flow chart of patient evolution with an anticipated organ donation approach during the 2013-2016 period. ICU, intensive care unit; OPO, organ procurement organization

During 2013-2016, the anticipated organ donation approach was initiated 135 times. A flow chart of patient evolution is provided in Figure 3. Among the 53 patients who died, 49 (92%) were actual donors.

## Discussion

Our study demonstrates that the anticipated organ donation approach increased the number of actual organ donors and the number of organs harvested, particularly kidneys. The number of potential donors increased by 28.3% between the two periods, whereas the national increase was 12.2%.^4^

The anticipated approach is initiated early after neuroaggression and is often implemented in the hospital emergency room after an initial clinical and paraclinical workup, including cerebral and transcranial Doppler ultrasonography. Identifying an untreatable cerebral lesion and making a decision after a multidisciplinary ethics discussion regarding the therapeutic intensity of palliative care will orient the patient toward the anticipated approach. Identifying early clinical and paraclinical data to predict subsequent brain death is essential for preventing unnecessary organ care. Retrospective studies have created scores to predict brain death, but no prospective studies have defined pertinent predictive criteria. de Groot et al.^7^ defined the principle of “imminent brain death” as “a mechanically ventilated, deeply comatose patient admitted to an ICU with irreversible catastrophic brain damage of known origin”.^3^ This condition requires a Glasgow coma score of 3 and progressive absence of at least three of six brainstem reflexes, or a score of E0M0B0R0 on the Full Outline of UnResponsiveness scale. Although this stage suggests a final evolution toward brain death, it is rarely detected in the emergency department before ICU admission. The condition is more frequently detected within 48 hours of ICU admission due to hematoma, ischemic edema, or hydrocephalus. No clinical argument has been established to predict the occurrence of brain death with sensitivity, and imagery data alone cannot predict it.^8, 9^ The evolution of the results of repeated neurological examinations, cerebral imagery, and transcranial Doppler ultrasonography over time, while eliminating confounding factors, enables clinicians to predict subsequent brain death. Although no usable predictive score is available in current clinical practice, data from the literature and successful experience have enabled us to select the criteria used in our protocol.

The anticipated organ donation approach is a proactive solution to organ donation^10, 11^ and involves all players from the emergency department to the ICU. Player involvement relies on the conviction that taking the time to confirm brain death can help resolve public health problems. de Groot et al.^7^ reported that professional practices have been modified in recent years. When brain death is anticipated, communication with families regarding the possibility of organ donation.

In our local OPO during the 2013-2016 period, 62% of the meetings with families occurred before the diagnosis of brain death was confirmed. Out of the 117 anticipated approaches performed, more than two-thirds of patients were admitted to the ICU, and over half of them donated organs. This overall proportion is close to that recorded in patients with brain death. Our approach increased the number of patients listed as brain dead in a population base, and the grafts confirmed our results. Experience with this technique acquired over time should result in an increase in the number of organs available for grafting. The next step is to fine-tune the predictive criteria and reduce errors secondary to procedure implementation. The refusal rate observed by our team was lower than that observed annually in France (29% vs. 33.7%).^4^

The interest of a standardized “anticipated approach” procedure is to enable all healthcare staff to identify potential donors beginning in the emergency room and to respect ethics and good practices during end-of-life situations. The algorithm for the care of untreatable catastrophic neurological patients is an aid for physicians, enabling them to resolve ethical problems between respecting the wishes of patients and public health given the shortage of grafts. The local OPO does not intervene in multidisciplinary discussions regarding the intensity of patient therapy but only intervenes after the decision to perform palliative care. In our experience, the local OPO is a real added value, as it improves the experience of the healthcare team and families by coordinating the data of different players. Potential patients were screened by specially trained personnel. These situations are emotionally stressful for families, and the healthcare team must be capable of proposing adapted and coherent care. Invasive treatment must not be continued if the patient is not ultimately brain dead or if there is a contraindication. This approach can reduce the number of available organs for patients on waiting lists. The positive evolution of the number of patients and organs harvested in our study should encourage teams to develop this anticipated approach. Implementation of this approach in hospitals requires the writing of a specific procedure that describes the steps involved in this type of care. The families are included in this donor approach.

The absence of proof of brain death is considered a failure by the healthcare team and family. Since 2014, France has authorized the harvesting of organs from patients who died of cardiac arrest after the discontinuation of active ICU therapy (Maastricht 3). Patients under 65 years of age who are included in the procedure can be donors despite the absence of proof of brain death. Therefore, an “ethical” continuity exists in these procedures that makes it possible to reduce the number of failures in the donor procedure.

The anticipated approach involves older patients who, for the most part, are stroke victims with multiple pathological histories. Most of these cases are “imperfect” donors, for whom the question of so-called “expanded criteria” organs. The use of grafts obtained from donors with expanded criteria is no longer in question,^12^ with the development of organ preservation techniques by machine perfusion^13^ enabling access to grafts for many patients who cannot be otherwise operated on.

### Study Limitations

We acknowledge several limitations of our study. First, we conducted a retrospective cohort study with limited available demographic and clinical data or reasons for family refusal. In addition, the initiation of the anticipatory approach was due to the medical team and their awareness of the detection and inclusion of these rapidly deteriorating patients for whom no care was previously offered, which may have underestimated our results. Finally, the criteria used to detect early a possible transition to a state of brain death within 48 hours were based on limited published data and the local experience of practitioners, which may have led to the exclusion of potential donors.

## Conclusion

The anticipated organ donation approach increases the number of organs available for transplantation, helping to address the public health issue of organ shortage. Implementing this approach requires a written protocol tailored to each hospital for the care of patients with untreatable catastrophic neurological injuries, as well as local OPO training and increased awareness among healthcare personnel. The anticipated approach respects patient autonomy by offering the option of organ donation or palliative care and aligns with the development of practices that facilitate communication between healthcare personnel and families. This procedure benefit from scientific advances in the early determination of predictive criteria for brain death and reinforces harvesting procedures after cardiocirculatory arrest.

## Ethics

**Ethics Committee Approval:** The study protocol consisted of a retrospective analysis of an anonymised database without any direct human involvement. In line with the French law prevailing at the time of the study, the protocol received approval (approval no.: 8.4.17, no.: 376) from the local ethics review board on April 8, 2017, at Ministere De La Defense/H.I.A. Sainte Anne.

**Informed Consent:** This study protocol consisted of a retrospective analysis of an anonymised database.

## Figures and Tables

**Table 1. Organ Donation Data for the Two Periods and the Anticipative Approach:** 

**Group**	**All patients**	**All patients**	**Evolution between the two periods**	**Only patients requiring the anticipated approach**
Period	2009-2012	2013-2016		2013-2016
Age	56.6	58.2		67.5
Potential organ donors	145	186	+28.3%	
Organ donors	91	119	+30.7%	49
Organs transplanted	323	485	+50.1%	194
Kidney	164	228	+39%	98
Liver	68	103	+51.5%	43
Heart	33	42	+27.3%	20
Lung	53	102	+92.5%	29
Pancreas	5	10	+100%	4
